# Second fertility-sparing surgery and fertility-outcomes in patients with recurrent borderline ovarian tumors

**DOI:** 10.1007/s00404-022-06431-5

**Published:** 2022-03-23

**Authors:** Lifei Wang, Qian Zhong, Qin Tang, Hongjing Wang

**Affiliations:** 1 Department of Gynecology and Obstetrics, West China Second University Hospital of Sichuan University, No.20, Section 3, Renmin South Road, Chengdu, Sichuan People’s Republic of China; 2 Key Laboratory of Birth Defects and Related Diseases of Women and Children, Sichuan University, Ministry of Education, Chengdu, People’s Republic of China

**Keywords:** Borderline ovarian tumors, Fertility sparing surgery, Radical surgery, Recurrence, Pregnancy outcome

## Abstract

**Background:**

At the time of recurrence, many borderline ovarian tumor (BOT) patients are still young with fertility needs. The purpose of this study is to evaluate the reproductive outcomes and recurrence rate of second fertility-sparing surgery (FSS) in women with recurrent BOTs.

**Methods:**

Seventy-eight women of childbearing age diagnosed with recurrent BOTs from November 2009 to 2020 whose primary treatment was FSS were included.

**Results:**

The FIGO stage I disease accounted for 46.2% and serous BOT accounted for 87.2% in the study group. Forty-seven patients underwent second FSS, and the remaining 31 underwent radical surgery (RS). Seventeen patients relapsed again after second surgery, but no malignant transformation and tumor-associated deaths were reported. Compared to FIGO stage I, the FIGO stage III tumors were more likely to relapse, but there was no statistical difference in pregnancy rate among patients with different stages. In the second FSS group, recurrence rate was higher in patients who underwent oophorocystectomy compared to patients with unilateral salpingo-oophorectomy (USO), but the pregnancy rate was similar. There was no significant difference in postoperative recurrence risk between USO and RS. The recurrence rate was not associated with operative route (laparoscopy or laparotomy), or lymphadenectomy, or postoperative chemotherapy. Among the 32 patients who tried to conceive, the pregnancy rate was 46.9% and live birth rate was 81.3%.

**Conclusion:**

Unilateral salpingo-oophorectomy is a safe procedure for FIGO stage I recurrent BOT patients with fertility needs, and can achieve a high postoperative pregnancy rate and live birth rate.

## Introduction

Borderline ovarian tumors (BOTs) are a subgroup of epithelial ovarian tumors, accounting for 10%–15% of all ovarian tumors. They are characterized by the histological features of malignant tumors, but without recognizable destructive interstitial invasion [1]. BOTs often have favorable prognoses. The 5-year survival rate for early stage disease is about 99%, and the 10-year survival rate is 97% [2].

The median age at the time of diagnosis of BOT is 45 years old, but 34% of the patients are under 40. Compared with epithelial ovarian cancer, BOT is mainly diagnosed in early stage [3]. Therefore, fertility-sparing surgery (FSS) is a common option, which can be unilateral or bilateral cystectomy, unilateral adnexectomy with or without contralateral cystectomy. For patients who have fulfilled their reproductive wishes, radical surgery (RS) including bilateral salpingo-oophorectomy with or without hysterectomy and incomplete or complete staging surgery are performed. Nonetheless, there is no obvious evidence supporting the necessity of systematic hysterectomy and lymphadenectomy [4].

It has been reported that the recurrence rate after FSS is higher than that of the RS [1]. At the time of recurrence, many patients are still young with fertility needs. However, it is still unclear whether a second fertility-sparing surgery can be performed [5]. The purpose of this retrospective study is to compare the recurrence and fertility results between conservative treatment and radical treatment in patients with recurrent BOTs.

## Methods

### Patients, pathology and treatment

From November 2009 to November 2020, 90 patients with recurrent BOTs were treated in the Department of Gynecology, West China Second University Hospital of Sichuan University, and their first operation was FSS. Among them, 78 patients were followed up by us. All diagnoses were confirmed by histopathology. The second FSS included oophorocystectomy, unilateral salpingo-oophorectomy with or without contralateral oophorocystectomy. The radical surgery included bilateral salpingo-oophorectomy with or without hysterectomy. An appendectomy was performed in all patients diagnosed with mucinous BOT. We used the Ovarian Cancer Classification of the International Federation of Obstetrics and Gynecology (FIGO 2014) to determine the stage of the BOTs. The second FSS was performed after a comprehensive evaluation at the request of the patients themselves. Patients were divided into 2 groups: the second FSS group and the RS group. Patients were followed up through direct telephone interviews and data were collected on disease recurrence and fertility outcomes. Because of its retrospectivity, this study was exempted from the ethical approval process. Patients expressed informed consent to the clinical data records and clinical research in the relevant clinical tumor registries, and voluntarily agreed to telephone follow-up.

### Statistical analysis

All statistical analyses were conducted by Statistical Product and Service Solutions (SPSS 26.0). The Kaplan–Meier method was used for univariate analysis of disease-free survival (DFS). Since no fatal event happened to the patient, we did not include any overall survival (OS) analysis in the manuscript. DFS was calculated on a monthly basis from the second operation to the recurrence date. Cox proportional hazard regression model is used to evaluate all parameters that are meaningful in univariate analyses. The multivariate adjusted odds ratios (OR) and 95% confidence intervals (CI) are expressed. All analyses are considered as hypothesis generation, and the *p* value < 0.05 was considered significant.

## Results

### Patients’ characteristics

**Fig. 1:**
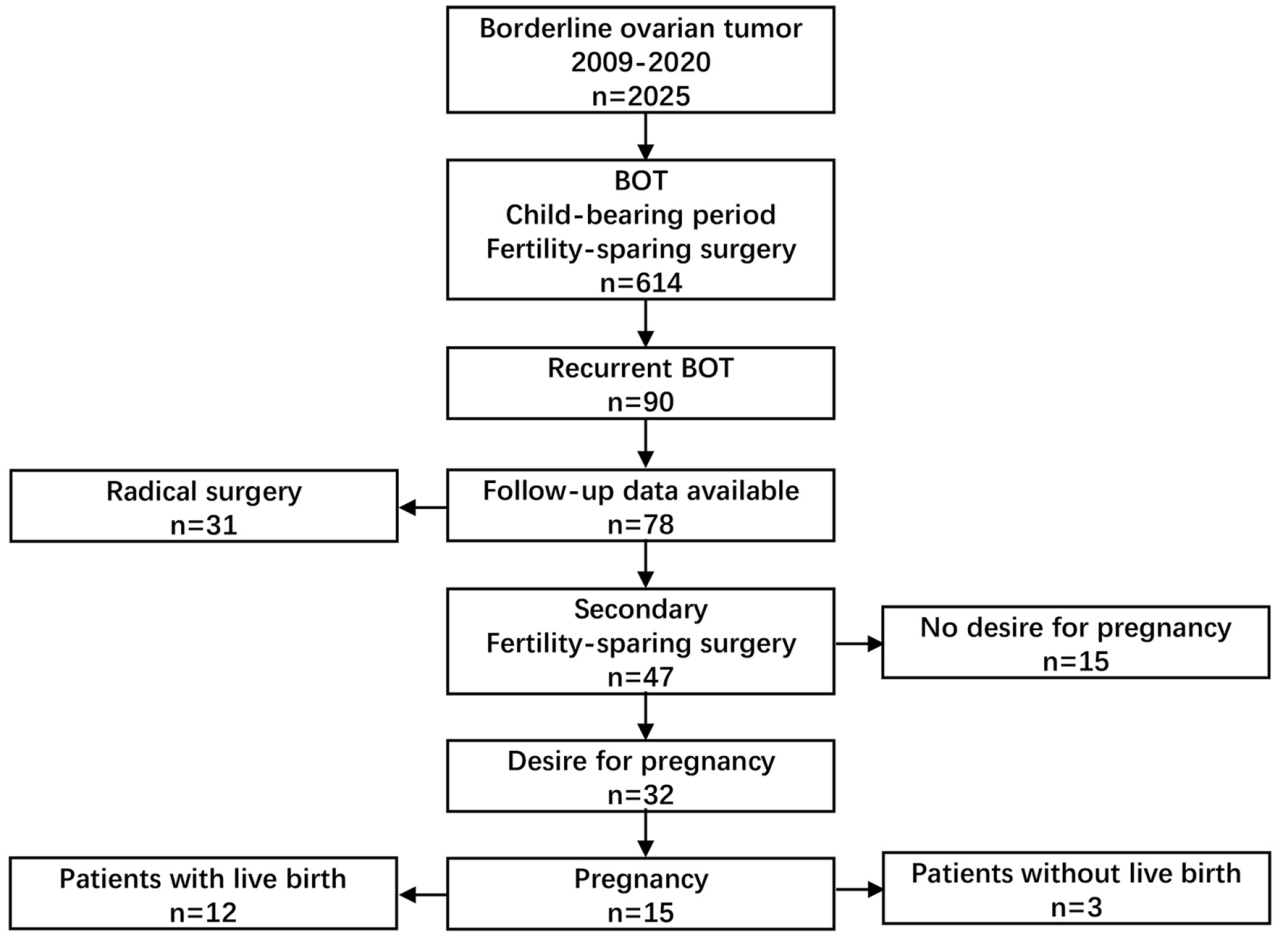
Flow chat of the recurrent BOT study

Seventy-eight patients with recurrent BOTs were included in the study (Fig. 1). Table 1 lists the characteristics of the patients in detail. The median age was 30 years (range 14–47 years), and the median follow-up time was 46.5 months (range 7–139 months). Most of the patients were diagnosed with FIGO stage I (46.2%) disease and serous BOT (87.2%). Twenty-three cases (33.8%) were of microcapillary type, and only one case had stromal microinvasion. Second operation was performed by laparotomy or laparoscopic surgery, accounting for 60.3% and 39.7% respectively. A total of 17 patients (21.8%) relapsed again, and all of them were still BOTs.

**Table 1:** Demographic characteristics of patients with recurrent borderline ovarian tumors

Characteristics	Patients (*n*, %)	Recurrence (*n*, %)
Age (median, range) (years)	30 (14–47)	
≤ 30	44 (56.4)	13 (29.5)
> 30	34 (43.6)	4 (11.8)
Size (median, range) (cm)	5.8 (2.3–22.0)	
≤ 5	23 (29.5)	4 (17.4)
> 5	55 (70.5)	13 (23.6)
CA-125 level (median, range) (U/mL)	23 (2.7–485.2)	
< 35	47 (60.3)	10 (21.3)
≥ 35	31 (39.7)	7 (22.6)
Histology		
Serous	68 (87.2)	17 (25.0)
With micropapillary pattern	23 (33.8)	6 (26.1)
Without micropapillary pattern	45 (66.2)	11 (24.4)
Mucinous	6 (7.7)	0 (0)
FIGO stage		
I	36 (46.2)	4 (11.1)
II	17 (21.8)	4 (23.5)
III	25 (32.0)	9 (36.0)
Fertility-sparing surgery (FSS)	47 (60.3)	15 (31.9)
Unilateral cystectomy	16 (34.0)	6 (37.5)
Bilateral cystectomy	9 (19.1)	5 (55.6)
Cystectomy and contralateral ovarian biopsy	2 (4.3)	2 (100.0)
Unilateral salpingo-oophorectomy (USO)	14 (29.8)	0 (0)
USO and contralateral cystectomy	3 (6.4)	1 (33.3)
USO and contralateral ovarian biopsy	3 (6.4)	1 (33.3)
Radical surgery (RS)	31 (39.7)	2 (6.5)
Bilateral salpingo-oophorectomy (BSO)	7 (22.6)	0 (0)
Hysterectomy and BSO	6 (19.4)	0 (0)
Staging surgery	18 (58.0)	2 (11.1)
Surgery approach		
Laparoscopy	31 (39.7)	7 (22.6)
Laparotomy	47 (60.3)	10 (21.3)
Lymphadenectomy		
Yes	21 (26.9)	3 (14.3)
No	57 (73.1)	14 (24.6)
Postoperative chemotherapy		
Yes	30 (38.5)	6 (20.0)
No	48 (61.5)	11 (22.9)

### Characteristics and outcomes of patients with second FSS

Forty-seven patients underwent second FSS. The median age of this subgroup was 28 years (range 14–39 years). Twenty-three (48.9%) patients suffered from FIGO stage I disease. Bilateral ovarian tumors occurred in 12 patients (25.5%). Table 1 summarizes the characteristics of certain surgical procedures. Sixteen (34.0%) of them underwent unilateral cystectomy, 9 (19.1%) underwent bilateral cystectomy, 2 (4.3%) underwent cystectomy plus contralateral ovarian biopsy, 14 (29.8%) underwent unilateral salpingo-oophorectomy (USO), 3 (6.4%) underwent USO plus contralateral cystectomy, 3 (6.4%) underwent USO plus contralateral ovarian biopsy.

#### Recurrence outcomes of second FSS

After a median follow-up of 39 months (range 7–137 months), 15 cases (31.9%) relapsed again, and all of them relapsed as BOTs. Median disease-free survival (DFS) was 31 months (range 7–110 months). Supplementary Table 1 describes the characteristics of the population with second recurrence.

#### Reproductive outcomes of second FSS

Thirty-two patients (68.1%) tried to conceive, among them 15 patients had at least one pregnancy. The median time interval from surgery to pregnancy was 18 months (range 3–39 months). The cumulative incidence of first pregnancy was 46.9%. Twelve women delivered, with the corresponding live birth rate being 81.3%. A total of 13 cases of live birth were reported, and 1 woman had 2 full-term pregnancies. One patient of primary infertility got pregnant after assisted reproductive technology (ART) and finally gave birth to a healthy baby (Table 2).

**Table 2:** Characteristics of patients who attempted to conceive after surgery

Characteristics	Pregnancy (*n*, %)	Live birth (*n*, %)
Age (years)		
≤ 30	13 (50.0)	11 (84.6)
> 30	2 (33.3)	1 (50.0)
BMI (Kg/M^2)		
< 24.0	10 (43.5)	9 (90.0)
≥ 24.0	5 (55.6)	3 (60.0)
Surgery type		
Laparoscopy	7 (43.8)	4 (57.1)
Laparotomy	8 (50.0)	8 (100.0)
Fertility-sparing surgery type		
Cystectomy	9 (47.4)	7 (77.8)
Salpingo-oophorectomy	6 (47.2)	5 (83.3)
FIGO stage		
I	9 (69.2)	6 (66.7)
II	1 (12.5)	1 (100.0)
III	5 (45.5)	5 (100.0)
Postoperative chemotherapy		
Yes	4 (50.0)	4 (100.0)
No	11 (45.8)	8 (72.7)
Way of pregnancy		
Spontaneous	14 (93.3)	10 (71.4)
ART	1 (6.7)	1 (100.0)

As shown in Table 3, the operation methods (laparoscopy and laparotomy) did not affect pregnancy rate (HR 0.99, 95% CI 0.36–2.75, *P* = 0.991). The pregnancy rate of patients with residual bilateral adnexa after operation was higher than that of patients with residual unilateral adnexa (60.0% vs 40.9%), but there was no statistical significance between the two groups (HR 1.53, 95% CI 0.54–4.30, *P* = 0.420).

**Table 3:** Results of univariate analyses of pregnancy rate

	Hazard ratio	95% CI	*P* value
Age (years)			
≤ 30 vs. > 30	1.65	0.37–7.31	0.511
BMI (Kg/M^2)			
< 24.0 vs. ≥ 24.0	0.81	0.28–2.37	0.700
FIGO stages			
I vs. II	9.02	1.13–71.86	0.038
I vs. III	2.63	0.87–7.90	0.085
II vs. III	0.22	0.03–1.86	0.163
Laparoscopy vs. laparotomy	0.99	0.36–2.75	0.991
Cystectomy vs. USO	0.95	0.34–2.66	0.919
Postoperative chemotherapy			
Yes vs. No	1.03	0.33–3.25	0.957

It is worth noting that there were two patients who relapsed during mid-pregnancy, but both of them gave birth successfully after the second FSS during pregnancy.

### Characteristics and outcomes of patients underwent RS

After recurrence, 31 patients (39.7%) received RS. The median age of these patients was 39 years (range 19–47 years). Twelve (38.7%) of the patients suffered from FIGO stage I disease, and 96.7% patients suffered from SBOT. Eight (25.8%) of patients underwent laparoscopic surgery. Bilateral salpingo-oophorectomy (BSO) were performed in 7 (22.6%) patients, hysterectomy and BSO were performed in 6 patients (19.4%), incomplete staging surgery were performed in 5 patients (16.1%) and complete staging surgery were in 13 patients (41.9%). In this subgroup, additional surgical procedures, such as omentectomy (in 58.1% patients) or pelvic and/or para-aortic lymphadenectomy (in 48.4% patients) were performed. Only two patients who underwent radical surgery relapsed again, and they were still BOTs.

### Risk factors for recurrence between second FSS group and RS group

#### Prognostic factors for second recurrence (univariate analysis)

According to Table 4, compared with only two recurrent cases (6.5%) in the RS group, the recurrence risk (RR) of patients who received second FSS was significantly higher, with 15 recurrent cases (31.9%) (hazard ratio [HR] 5.66; 95% CI 1.29–24.76; *P* = 0.021). The RR was also significantly higher in younger patients (≤ 30 vs. > 30 years; HR 3.68, 95% CI 1.06–12.82, *P* = 0.041) and in patients with higher FIGO stages (FIGO I vs. III; HR 0.29, 95% CI 0.09–0.94, *P* = 0.039). In the second FSS group, RR was significantly higher in patients who underwent oophorocystectomy compared to unilateral salpingo-oophorectomy (HR 6.11, 95% CI 1.38–27.15, *P* = 0.017).

**Table 4:** Results of univariate analyses of recurrence rate

	Hazard ratio	95% CI	P value
Age (years)			
≤ 30 vs. > 30	3.68	1.06–12.82	0.041
CA125 level			
Abnormal vs. normal	0.92	0.35–2.42	0.865
Recurrence site(s)			
Unilateral vs. bilateral	0.56	0.21–1.47	0.240
Micropapillary pattern			
Yes vs. No	0.88	0.33–2.38	0.801
FIGO stages			
I vs. II	0.47	0.12–1.87	0.281
I vs. III	0.29	0.09–0.94	0.039
II vs. III	0.61	0.19–1.99	0.413
Laparoscopy vs. laparotomy	1.10	0.42–2.89	0.846
Lymphadenectomy			
Yes vs. No	0.56	0.16–1.93	0.355
FSS vs. RS	5.66	1.29–24.76	0.021
FSS (FIGO I) vs. RS (FIGO I)	1.82	0.19–17.48	0.605
FSS (FIGO II) vs. RS (FIGO II)	1.65	0.17–15.87	0.665
FSS (FIGO III) vs. RS (FIGO III)	99.55	0.40–24,642.66	0.102
Cystectomy vs. USO	6.11	1.38–27.15	0.017
USO vs. RS	1.57	0.22–11.16	0.651
Postoperative chemotherapy			
Yes vs. No	0.87	0.32–2.36	0.787

There were no statistical significance in RR between SBOT with or without micropapillary pattern (HR 0.88, 95% CI 0.33–2.38, *P* = 0.801), laparoscopy or laparotomy (HR 1.10, 95% CI 0.42–2.89, *P* = 0.846), lymphadenectomy or not (HR 0.56, 95% CI 0.16–1.93, *P* = 0.355), postoperative chemotherapy or not (HR 0.87, 95% CI 0.32–2.36, *P* = 0.787), as well as unilateral salpingo-oophorectomy or RS (HR 1.57, 95% CI 0.22–11.16, *P* = 0.651).

#### Multivariate cox-regression analysis

Multivariate Cox- regression analysis (adjusted to the 3 most important variables: age, FIGO stage, FSS and RS) showed that FIGO stage III (HR 4.73, 95% CI 1.44–15.52, *P* = 0.012) and FSS (HR 12.35, 95% CI 1.56–97.66, *P* = 0.017) were independent prognostic factors for second recurrence, but age had no statistical significance (≤ 30 vs. > 30 years; HR 1.18, 95% CI 0.32–4.39, *P* = 0.803).

## Discussion

Since quite a number of recurrent BOT patients are of reproductive age, second fertility sparing surgery has become an increasingly important issue when discussing treatment options [6]. In this study, patients with recurrent BOTs in childbearing age who relapsed after the first conservative operation were followed up. Based on fertility needs and recurrence risk, 60.3% of these patients underwent a second fertility-sparing surgery and the rest 39.7% underwent radical surgery. Recurrent BOT is usually associated with a good prognosis, especially in FIGO stage I disease, and the survival data from previous studies is excellent [7]. Therefore, conservative surgery for these patients has become a trend. The purpose of this study is to analyze the recurrence risk and pregnancy outcome in patients with recurrent BOTs who received second FSS. To achieve this goal, we compared the risk of relapse in patients who received second FSS with those who received radical surgery.

In our cohort, the recurrence rate after second FSS was 31.9%, while the RR after radical surgery was only 6.5%. Other studies have also reported that recurrence rate after second FSS was between 25.0% and 42.3% [5, 7], and the RR after radical surgery was 27.3% [8]. However, since there were no invasive recurrences and disease-related deaths in patients with second FSS, this treatment option can be considered. The pregnancy rate after second FSS was 46.9%, close to the data reported by Catherine Uzan et al.(50%) [7], and Jia, SZ et al.(47%) [9]. Univariate analysis was used to determine factors related to high fertility results, but because of the limited number of cases, we didn't find anything with statistical significance.

Zilliox, M. et al. have shown the feasibility of treating BOTs surgically during pregnancy [10]. Study has also demonstrated that "expectant treatment" could be a safe choice for recurrent BOTs found in pregnancy [11]. Two patients in our study were pregnant at the time of the second recurrence, and they underwent laparoscopic operation under general anesthesia at mid-pregnancy. Both of them delivered successfully after surgery.

Kurman, R.J, et al. have reported that the development and progression of high-grade ovarian cancer and low-grade ovarian tumor including BOT follow two different molecular pathways and have different biological behaviors [12]. In our study, the pathological type of all recurrent diseases remained BOTs and no malignant transformations were found, which is consistent with the "dualistic model" of ovarian malignancy mentioned above. Studies have shown that the malignant transformations often occur at the age of 41–57 years [4, 13]. Our patients were in their childbearing years with a median age of 30 years, which may be the reason for the absence of malignant transformation. This may partly explain why although other studies have shown that the presence of microcapillaries increases the risk of recurrence and malignant transformation in BOTs [14, 15], there was no statistical difference in the risk of recurrence between patients with and without microcapillary pattern in our study. In addition, the favorable disease-free survival in patients with mucinous BOTs in our research is consistent with the result in other reports [16, 17].

Although laparoscopic approach was thought to be associated with higher rate of cyst rupture and incomplete staging [18], many studies have shown that the choice of laparoscopy or laparotomy does not affect the recurrence and prognosis of patients with BOTs [19–21], which is consistent with our findings.

In the second FSS group, oophorocystectomy significantly increased the risk of recurrence compared with salpingo-oophorectomy, and there was no statistical difference in postoperative pregnancy rate between the two procedures. Therefore, the choice of oophorocystectomy should be made with great caution [22, 23]. Our study also showed that neither lymphadenectomy nor postoperative chemotherapy had any positive effect on recurrence and pregnancy outcomes, which is consistent with previous studies [24, 25]. Furthermore, there was no significant difference in recurrence rate between adnexectomy and hysterectomy with adnexectomy, which is consistent with the findings of Ouldamer, L, et al. [26] and Matsuo, K, et al. [24]. In a similar vein, the study by Mandelbaum RS et al. suggested that preservation of the uterus ovary in patients with early BOT may be associated with improved overall survival compared with preservation of the ovary alone [27].

FIGO stage has been described as one of the most important prognostic factors for BOTs [7, 8]. Our multivariate analysis revealed that FIGO stage III is an independent risk factors for recurrent BOTs.

In conclusion, second FSS in FIGO stage I is a safe operation for patients with reproductive needs, and can achieve high pregnancy and live birth rates after operation. For patients with more advanced FIGO stages, the second FSS should be weighed against pros and cons. Compared to unilateral salpingo-oophorectomy, the oophorocystectomy had statistically higher RR without extra benefit in pregnancy outcome. The risk of recurrence after RS and unilateral salpingo-oophorectomy was comparable and not statistically different. Patients with recurrent BOTs may not benefit from hysterectomy, lymphadenectomy, or postoperative chemotherapy.

## Data Availability

All data and materials are available.
